# Takeaways from Mobile DNA Barcoding with BentoLab and MinION

**DOI:** 10.3390/genes11101121

**Published:** 2020-09-24

**Authors:** Jia Jin Marc Chang, Yin Cheong Aden Ip, Chin Soon Lionel Ng, Danwei Huang

**Affiliations:** 1 Department of Biological Sciences, National University of Singapore, 16 Science Drive 4, Singapore 117558, Singapore; ycip@u.nus.edu (Y.C.A.I.); lionel.ngcs@gmail.com (C.S.L.N.); 2 Tropical Marine Science Institute, National University of Singapore, 18 Kent Ridge Road, Singapore 119227, Singapore

**Keywords:** cytochrome c oxidase subunit I (COI), marine biodiversity, metazoa, next-generation sequencing (NGS), Oxford Nanopore Technologies (ONT), portable sequencing

## Abstract

Since the release of the MinION sequencer in 2014, it has been applied to great effect in the remotest and harshest of environments, and even in space. One of the most common applications of MinION is for nanopore-based DNA barcoding in situ for species identification and discovery, yet the existing sample capability is limited (*n* ≤ 10). Here, we assembled a portable sequencing setup comprising the BentoLab and MinION and developed a workflow capable of processing 32 samples simultaneously. We demonstrated this enhanced capability out at sea, where we collected samples and barcoded them onboard a dive vessel moored off Sisters’ Islands Marine Park, Singapore. In under 9 h, we generated 105 MinION barcodes, of which 19 belonged to fresh metazoans processed immediately after collection. Our setup is thus viable and would greatly fortify existing portable DNA barcoding capabilities. We also tested the performance of the newly released R10.3 nanopore flow cell for DNA barcoding, and showed that the barcodes generated were ~99.9% accurate when compared to Illumina references. A total of 80% of the R10.3 nanopore barcodes also had zero base ambiguities, compared to 50–60% for R9.4.1, suggesting an improved homopolymer resolution and making the use of R10.3 highly recommended.

## 1. Introduction

The practice of DNA barcoding—involving the generation of standardized genetic markers that, when matched to databases, allow for species identification—was first popularized by Hebert et al. (2003) [1]. Since then, the field of DNA barcoding has evolved and expanded considerably beyond just species identification [2,3,4,5] to include species discovery, population genetics, and phylogenetics [6,7,8,9,10,11,12,13]. This rapid growth in DNA barcoding capabilities has occurred as a result of advancements in sequencing technologies. For instance, the rise of second-generation sequencers (e.g., Illumina) has greatly enhanced our ability to produce DNA barcodes in larger volumes (vis-à-vis Sanger sequencing) while maintaining high accuracy and low costs [14,15,16,17]. However, one of the limitations of second-generation sequencing technologies is that the DNA barcoding process and its associated technologies largely remain spatially confined to specialized laboratory settings.

The development of the MinION sequencer by Oxford Nanopore Technologies (ONT) was thus significant for nucleic acid sequencing as it quickly materialized the concept of portable sequencing. Its release was game-changing for several reasons, though most notably for its compact size and portability, as well as its ability to generate data in real time [18]. Since then, the MinION sequencer has been adopted to great effect in some of the remotest and harshest of environments [19,20,21,22], including the International Space Station [23]. Nanopore sequencing has also featured prominently in the monitoring of disease outbreaks such as Ebola [24,25,26], and more recently in the detection of SARS-CoV-2 [27,28,29]. However, portable DNA barcoding for biodiversity identification and discovery remains limited in application and is restricted to fairly small sample sizes (*n* ≤ 10) [20,21,30,31,32]. We thus sought to assemble a mobile sequencing workflow that would enhance the capacity for in situ DNA barcoding.

**Figure 1:**
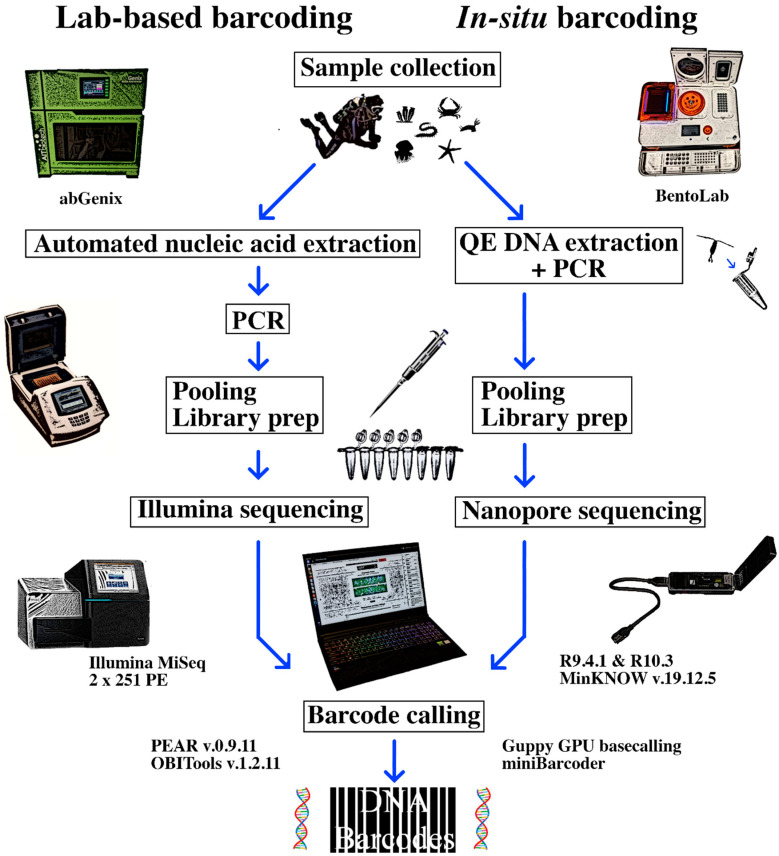
Schematic representation of our in situ field sequencing workflow (**right**), from sample to sequence, compared with a typical laboratory-based next-generation sequencing barcoding workflow (**left**).

To achieve this, we combined the use of the BentoLab (https://www.bento.bio/) with the MinION sequencer (Figure 1). The BentoLab is a suitcase-sized, mobile genetics setup that contains essential laboratory instruments such as a 32-well thermocycler, a 6-well centrifuge, and a gel electrophoresis dock [33]. We chose the BentoLab over other portable laboratory devices like the miniPCR^TM^ (8-wells) as our proposed workflow was reliant on having a higher thermocycling capacity (see Methods). The BentoLab–MinION combination for portable sequencing is not new [32,34]. One study explored the rapid characterization of single-nucleotide polymorphisms for forensics [34], while another demonstrated the feasibility of in situ DNA barcoding of nematodes [32]. Here, our proposed pipeline is designed to comfortably handle larger sample sizes and more diverse fauna through the use of degenerate metazoan primers. We selected the miniBarcoder as our nanopore barcoding pipeline for its well-established high-throughput capacity [35,36,37], which would open up opportunities for non-academic or non-research-based agencies to employ DNA barcoding for their own applications. We first trialed the BentoLab processes ex situ using samples that were routinely obtained from various intertidal and subtidal surveys. These collections were part of an ongoing effort to document the marine fauna in Singapore as well as to grow the local biodiversity knowledge base and barcode databases [38,39]. We then brought the sequencing setup out to the field and performed the entire sample-to-sequence workflow out at sea to demonstrate its utility. We also took the opportunity to evaluate the performance of the newly released R10.3 flow cell for DNA barcoding. This new version features nanopores with dual-reader heads for improved resolution of homopolymer information, and promises more accurate consensus reads. We were thus interested in comparing the performance of DNA barcodes generated from the R10.3 flow cells with the more established R9.4.1 chemistry.

## 2. Materials and Methods

### 2.1. Sample Collection

Metazoan specimens were collected opportunistically from ten coral reef sites across Singapore from 2017 to 2019, either via intertidal surveys or subtidally via SCUBA. Collections were authorized by the National Parks Board (permit number NP/RP15-088), and samples were carefully treated according to NUS Institutional Animal Care and Use Committee (IACUC) guidelines (IACUC Protocol B15-1403) during the collection and vouchering process. During the vouchering phase, samples were grouped into phylum/class based on morphology. This was to facilitate downstream amino acid correction (see Section 2.5.), as well as morphology–barcode identity congruence checks. Voucher specimens were imaged using the Canon EF 100 mm f2.8/L IS USM macro lens on an EOS 750D.

### 2.2. Illumina NGS Barcoding as Reference

Genomic DNA extractions were either carried out using phenol:chloroform:isoamyl-alcohol (25:24:1) phase separation [39], or via the abGenix^TM^ automated DNA and RNA extraction system (AITbiotech Pte Ltd., Singapore) with Animal Tissue Genomic DNA Extraction kits according to the manufacturer’s protocols. All 151 samples were processed individually regardless of extraction method.

We amplified the 313-bp region of the mitochondrial cytochrome oxidase subunit I (COI) locus, using the mlCOIintF: 5′-GGW ACW GGW TGA ACW GTW TAY CCY CC-3′ [40] and LoboR1: 5′-TAA ACY TCW GGR TGW CCR AAR AAY CA-3′ [41] primer combination. The primer pair was chosen due to the high amplification success of marine fauna [39], and was also comparatively cheaper [15,37] than the conventional metabarcoding primer pair jgHCO2198 [42] and mlCOIintF (Supplementary Table S1). PCR primers were each tagged with unique 8-bp barcode tags on the 5′ end to allow for convenient downstream demultiplexing [15], and we ensured that forward and reverse tag combinations were unique to each specimen. Each PCR reaction mix comprised 2 µL of template DNA, 2 µL each of 10 µM 8-bp tagged primer, 1 µL of bovine serum albumin (BSA; 1 mg/mL), 1 µL of magnesium chloride, and 12.5 µL of GoTaq^®^ Green Master Mix (Promega), and was topped up to 25 µL with nuclease-free water. A step-up thermal cycling profile was used: 94 °C for 60 s; 5 cycles of 94 °C for 30 s, 48 °C for 120 s, 72 °C for 60 s, followed by 30 cycles of 94 °C for 30 s, 54 °C for 120 s, 72 °C for 60 s, and a final extension for 5 min at 72 °C. Amplification success was verified on 2% gels stained with GelRed (Cambridge Bioscience).

PCR amplicons were pooled based on gel band intensity and cleaned using 1.1× Sera-Mag^TM^ Magnetic SpeedBeads^TM^ (GE Healthcare Life Sciences) in 18% polyethylene glycol-8000 (PEG-8000) buffer (1 M NaCl, 10 nM Tris-HCl, 1nM EDTA, pH 8). We then prepared PCR-free libraries using the NEBNext^®^ Ultra^TM^ II DNA library prep kit (New England Biolabs), but with TruSeq DNA Single Indexes (Set B, Illumina), following the manufacturer’s instructions up to the adapter ligation step. Libraries were cleaned using the same 1.1× Sera-Mag PEG suspension, and sequenced in batches over two Illumina MiSeq lanes (251 × 251-bp) at the Genome Institute of Singapore. Note that each batch utilized only ~10% of each sequencing lane.

We followed the modified bioinformatic pipeline based on Sze et al. (2018) [43] and Leveque et al. (2019) [44], where we used PEAR v0.9.11 [45] to merge paired-end reads, and OBITools v1.2.11 [46] for demultiplexing and further downstream processing of assembled reads. We considered Illumina barcodes valid if (1) the dominant read sequence for the sample had a minimum 50× read coverage, and (2) if the dominant read sequence was at least five times more abundant than the next most dominant read sequence assigned to that sample [15,47]. Finally, we performed a translation check of Illumina barcodes on Geneious R11 v11.1.5 [48] to ensure there were no internal stop codons.

### 2.3. Laboratory BentoLab Extraction and Amplification

In preparation for the field sequencing phase, we first tested extractions and gene amplification with the BentoLab in the laboratory. We used QuickExtract^TM^ (Lucigen; heron referred to as “QE”), a DNA extraction solution which requires only incubation with a heat source to produce PCR-ready genomic DNA. This can be easily supplied by the thermocycling component of the BentoLab, thus making it a potentially convenient method of DNA extraction in situ. The QE solution has been used extensively on insects [35,36,49,50,51] as well as zooplankton [52] but only on a handful of marine macrofauna [39]. We tested the QE-based protocol on the BentoLab for the same group of samples prior to field sequencing. Tissue subsamples were immersed in 10 µL of QE solution, and reactions were incubated at 65 °C for 15 min, followed by 98 °C for 2 min [35]. The QE products were then diluted 10× prior to PCR with nuclease-free water, following the manufacturer’s recommendation, to reduce PCR inhibition.

Gene amplification was performed using the same primer pair described above. For MinION-based barcoding, however, the primers were tagged with 13-bp tag sequences (instead of the 8-bp tagged primers used previously for Illumina sequencing) to account for the higher sequencing error rate in nanopore sequencing [53], while still allowing for accurate sample demultiplexing downstream [35]. Our 25 µL PCR reaction mix was altered to: 2 µL of template DNA, 1 µL each of 10 µM 13-bp tagged primer, 2 µL of BSA (1mg/mL), and 12.5 µL of GoTaq^®^ Green Master Mix (Promega), and topped up with nuclease-free water. We replaced magnesium chloride with more BSA to better neutralize potential PCR inhibitors that might be present in the extracts [54]. We also took this opportunity to test if a shortened cycling profile would be feasible. The thermal cycling profile used was 94 °C for 60 s; 5 cycles of 94 °C for 30 s, 48 °C for 45 s, 72 °C for 45 s, followed by 30 cycles of 94 °C for 30 s, 55 °C for 45 s, 72 °C for 45 s, and a final extension for 3 min at 72 °C. Gene amplification success was likewise verified on 2% agarose gels. We pooled the amplicons by gel band intensity, taking 5 and 7 µL for bright and faint to no observed gel bands, respectively. The amplicon pool was cleaned with 1.1× AMPure XP magnetic beads (Beckman Coulter) and stored at −30 °C till the field sequencing phase.

### 2.4. Field Sequencing with BentoLab and MinION

**Figure 2:**
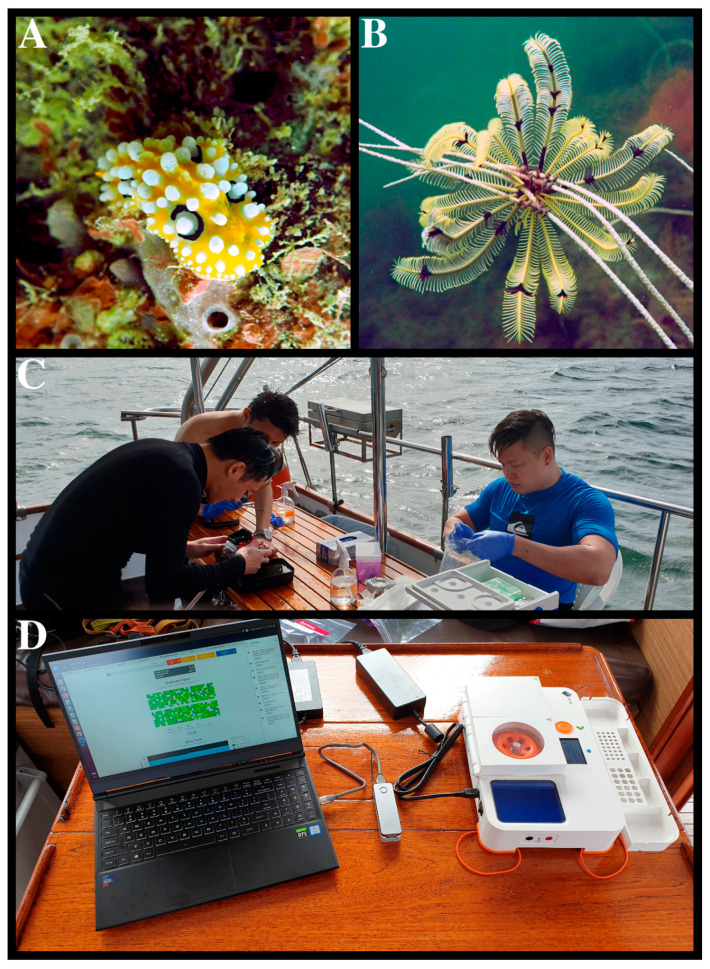
DNA barcoding performed in situ at Sisters’ Islands Marine Park, Singapore on 15 July 2020. Examples of samples collected via SCUBA: (**A**) HS0171, *Phyllidia ocellata*; (**B**) HS0179, *Cenometra bella*. (**C**) Samples were processed onboard and (**D**) barcoded using the BentoLab and MinION.

We performed field extraction, PCR, and sequencing as a proof-of-concept demonstration that the entire workflow was field-ready. Here, we assembled an in situ barcoding workflow involving the BentoLab, MinION sequencer, and a laptop computer (Intel^®^ core i7-9750H; Figure 1). We tested the system out at sea onboard a dive vessel moored off Sisters’ Islands Marine Park, Singapore on 15 July 2020, and documented the process from sample to sequence (Figure 2).

During the field trip, thirty-one fresh invertebrate metazoan samples were collected via SCUBA. Collections were authorized by the National Parks Board (permit number NP/RP20-037). Samples were subsampled onboard the diving vessel. All 31 samples, including one negative control, were extracted and gene-amplified using the BentoLab with the same methods described above (see Section 2.3.), but with minor adjustments. We increased the volume of QE per reaction to 20 µL, and decreased the total number of PCR cycles to 30. We ensured that the tag combinations used in the field PCR step did not overlap with the tagged amplicons generated at the home laboratory. Liquids were mixed by flicking the tubes or pipetting by hand. We also did not check for amplification success on agarose gel, and proceeded to pool the PCR products (taking 5 µL each) together with the amplicons generated ex situ for the bead clean-up using 1.1× AMPure XP magnetic beads (Beckman Coulter). Drying of the magnetic pellets was performed using a phone-powered mini fan. The final amplicon pool was quantified using a Qubit 3.0 Fluorometer with the Qubit dsDNA BR assay kit (ThermoFisher Scientific, Waltham, MA, USA).

We prepared a MinION library onboard using the Ligation Sequencing Kit (SQK-LSK109), with the following modifications: (1) end repair and dA-tailing reactions were incubated in the BentoLab at 20 °C for 15 min, followed by 65 °C for 15 min, and (2) ligation reactions were similarly incubated for 15 min at 20 °C. This undoubtedly increased the library preparation time, but we noted improved library success with the protocol changes [37]. Bead clean-ups were performed after end repair and adapter ligation. The library was sequenced on a fresh R9.4.1 flow cell, and left to run on a laptop (MinKNOW v.19.12.5) for ~50 min.

As we had exhausted the amplicon pool during library preparation for the first flow cell, we re-pooled the amplicons and prepared a second library for sequencing on a fresh R10.3 flow cell on the same laptop back at the laboratory. No changes were made to the reaction conditions. We monitored the sequencing progress and ended the run when an approximately same number of reads was generated as the R9.4.1 dataset. Run time for R10.3 lasted 2 h 30 min.

### 2.5. MinION Bioinformatics

For both sets of MinION raw reads, we performed GPU basecalling via Guppy v4.0.14 + 8d3226e. For the R9.4.1 flow cell, we generated two datasets, one produced using the fast basecalling model (“Fast”), and the other via the high-accuracy (“HAC”) model. The latter basecalling model produces higher single read accuracy, but is computationally more intensive than the former, and hence slower. We sought to investigate if the basecalling model had an impact on MinION barcodes generated from an error correction pipeline like miniBarcoder. For the R10.3 dataset, we started with two raw datasets. The first dataset was subsampled to the same run time as on R9.4.1 (50 min; hereon referred to as “ST”), while the second dataset had approximately the amount of reads generated (~1 million) as the R9.4.1 flow cell (heron known as “SR”). For both R10.3 read sets, we likewise performed basecalling using the Fast and HAC models. All six instances of basecalling were performed using the same settings, and we also noted the time taken for each instance (Supplementary Table S2).

We then performed MinION barcode calling using the miniBarcoder pipeline [35]. First, we used the miniBarcoder.py script to generate preliminary MAFFT barcodes via an alignment consensus approach. Briefly, the python script employed glsearch36 [55] to search for primer sequences in order to retrieve flanking tag sequences (Supplementary File S1), which were then used to bin reads into respective samples, before MAFFT v7.470 [56] was applied at the sample level for alignment of binned reads to call a majority consensus, or the “MAFFT barcode” [36]. Any resulting MAFFT barcodes that had <10× read coverage and >1% ambiguous bases called as Ns were discarded. We then applied racon_consensus.py to map the raw reads back to the MAFFT barcode using Graphmap v0.5.2 [57] before generating consensus sequences using RACON v1.4.13 [58] to yield “RACON barcodes” [36]. We subsequently used publicly available GenBank sequences (*nt* database updated 8 July 2020) for amino acid correction [36] of the MAFFT and RACON barcodes to yield “MAFFT + AA” and “RACON + AA” barcodes, respectively. As our sample set consisted of fauna from various phyla, the appropriate genetic code (option -g) was applied in the correction process [37]; we used code 2 for Actinopterygii, code 4 for Cnidaria and Porifera, code 9 for Echinodermata, Hemichordata, and Platyhelminthes, code 13 for Ascidiacea, and code 5 for the remaining invertebrates. We also varied the namino parameters from 1 to 3 [35]. The final step was to align the corrected MAFFT+AA and RACON + AA barcodes and call a strict consensus (using consolidate.py) to produce “consolidated barcodes” [36]. We used SeqKit v0.12.1 [59] and GNU Parallel [60] to accelerate barcode calling (see Supplementary File S2 for UNIX script for automating miniBarcoder). All MinION barcode calling steps were executed locally on the dedicated field sequencing laptop. The entire miniBarcoder pipeline took ~25–30 min for each dataset totaling 188 amplicons (179 samples + 9 negatives).

### 2.6. Assessing MinION Barcode Accuracy and Quality

We first subjected the Illumina and MinION barcodes to a contamination check. For the MinION barcodes, we used the MAFFT barcode dataset as it was the largest, and correspondingly filtered the other types of MinION barcodes of detected contaminants [35,37]. We performed a blastn search (NCBI BLAST+ v2.9.0; [61]) on the same offline *nt* database (-evalue 1e^−6^, -max_target_seqs 10, -perc_identity 70), and blast results were parsed through readsidentifier v1.0 (≥80% identity and 250-bp overlap [62]) to obtain taxonomic identities. We only accepted species-level identities for barcode matches ≥97% [1,2,39]. The taxonomic identities from readsidentifier were then compared against morphological classifications made during the sample vouchering process, and any incongruence was flagged for further voucher examination to preclude misidentifications. If a pre-sorting error was deemed unlikely, the barcodes were subsequently removed from the dataset. Any barcode that matched any non-metazoan sequence was also excluded from downstream analyses.

We then evaluated the MinION barcode datasets based on two criteria: (1) sequencing accuracy, and (2) barcode ambiguity. Sequencing accuracy is defined as the proportion of perfectly matched bases to the total number of bases compared, while barcode ambiguity refers to the proportion of ambiguous bases called as Ns that persists after amino acid correction [36]. These Ns were introduced to preserve the reading frame [35,36], and served to correct the sequencing errors in homopolymeric regions [63,64,65]. As a point of reference for sequencing accuracy, we used the barcodes generated via Illumina (Supplementary File S3) as the sequencing technology has already been proven to be highly accurate [66,67]. Our goal was to find the flow cell chemistry and basecalling model that scored high and low on sequencing accuracy and barcode ambiguity, respectively. We used the supplied assess_uncorrbarcodes_wref.py and assess_corrbarcodes_wref.py scripts [36]; the former utilized dnadiff v1.3 [68] to compare uncorrected barcodes against Illumina references, while the latter utilized MAFFT for alignment and pairwise comparisons of the corrected barcodes with Illumina ones [35,36]. Any MinION barcode that differed from its Illumina reference by >3% was deemed erroneous and flagged for removal. Barcode ambiguity was assessed using the measure_ambs.py script [36], and visualized as boxplots on R3.4.3 (R Core Team, 2017) using ggplot2 [69]. We then compared results across all six datasets to select the best performing MinION barcode dataset.

With the chosen MinION barcode dataset, we examined samples that failed the Ns-filtering step—these usually have a high number of Ns in the MAFFT barcode sequence, which in turn suggested the presence of contaminant reads [36]—and determined if they could be rescued. We approached these failed samples in a manner analogous to Ho et al. (2020) [70], which was to treat these samples as small-scale metabarcoding pools, except in this case, the sample sequences were mixed with contaminant reads. We took all the binned reads in each failed sample and subjected them to blastn against the same *nt* database and parsed the matches through readsidentifier v1.0 [62] to obtain their taxonomic identities. Barcode calling for the sample was repeated using only the reads that matched the morphological assignment of the voucher, and only if the retained read count was still ≥10. Only four samples (HS0019–20, HS0044, and HS0157) were re-examined this way. Finally, we performed objective clustering to collapse the DNA barcodes into molecular operational taxonomic units (MOTUs), i.e., putative species units, based on uncorrected *p*-distances [71,72]. We performed the clustering at 2–4% to check for MOTU stability. A final blastn was conducted, and taxonomic identities were obtained by parsing the best matches through readsidentifier.

## 3. Results

### 3.1. Marine Faunal Diversity

**Figure 3:**
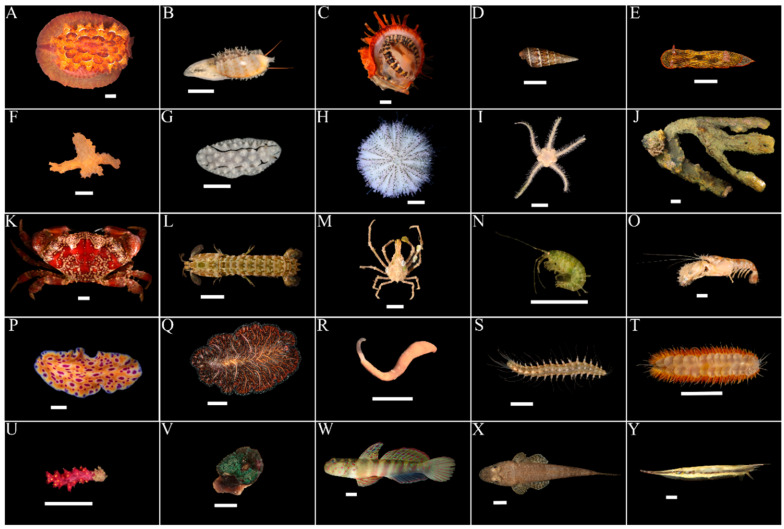
Representatives of sampled phyla in this study. Scale bars represent 1 cm. Phylum Mollusca: (**A**) HS0097, *Pleurobranchus forskalii*; (**B**) HS0074, *Erronea ovum*; (**C**) HS0147, *Spondylus* sp.; (**D**) HS0112, *Batillaria zonalis*; (**E**) HS0009, *Chromodoris lineolata*; (**F**) HS0148, *Crosslandia daedali*; (**G**) HS0067, *Phyllidiella rudmani*. Phylum Echinodermata: (**H**) HS0133, *Salmacis sphaeroides*; (**I**) HS0071, Ophiuroidea sp. Phylum Porifera: (**J**) HS0050, *Pseudoceratina* sp. Phylum Arthropoda: (**K**) HS0038, *Lophozozymus pictor*; (**L**) HS0042, *Gonodactylellus viridis*; (**M**) HS0096, Majoidea sp.; (**N**) HS0087, Amphipoda sp.; (**O**) HS0043, Alpheidae sp. Phylum Platyhelminthes: (**P**) HS0069, *Pseudoceros* sp 6; (**Q**) HS0039, *Pseudobiceros bedfordi*. Phylum Sipuncula: (**R**) HS0014, *Phascolosoma* sp. Phylum Annelida: (**S**) HS0143, *Leocrates* sp.; (**T**) HS0076, Polynoidae sp. Phylum Cnidaria: (**U**) HS0072, Alcyonacea sp.; (**V**) HS0145, *Discosoma* sp. Phylum Chordata: (**W**) HS0031, *Cryptocentrus leptocephalus*; (**X**) HS0134, Platycephalidae sp.; (**Y**) HS0064, *Aeoliscus strigatus*.

We collected 144 samples between August 2017 and January 2019 from ten coral reef sites across Singapore, representing 11 phyla (Figure 3). We also included seven samples from a previous study [39], for which we were unable to obtain DNA barcodes. The sample size for the laboratory trial was 151. Together with 31 samples collected on the field sequencing day, the total sample size for this study was 182. Samples for which whole vouchers were collected have been deposited in the Zoological Reference Collection at the Lee Kong Chian Natural History Museum, Singapore (Supplementary File S4).

### 3.2. Gene Amplification

For the laboratory-based BentoLab trial, there were 115 samples for which we had sufficient tissue subsamples to re-extract with QE solution, followed by PCR. Gel bands were observed for 69 samples (60%). We also repeated the MinION-based PCR for the remaining 33 sample extracts with insufficient tissue and obtained gel bands for 28 of them (~85%). Three samples (HS0043, HS0076, and IP0310) did not have a tissue subsample for QE re-extraction or genomic DNA for re-PCR (total for laboratory phase = 115 + 33 + 3 = 151 samples). Amplification success for the laboratory trial was ~66% on average (69 + 28 = 97 bands, out of 148 samples). For the field sequencing phase, an additional 31 samples were collected and subjected to QE-based DNA extraction on the BentoLab. While we did not run the gel check in situ to save time, a postliminary amplification check on agarose back at the laboratory revealed 20 observable gel bands.

### 3.3. Barcode Calling

We obtained 906,318 reads for the two Illumina libraries of 150 samples, which yielded 123 sequences; 116 sequences were retained after contamination and stop codon translation checks (Supplementary File S3). Read depths for our Illumina barcodes ranged between 59 and 33,795 per sample.

For our MinION-based barcoding approach, the R9.4.1 flow cell was run for ~50 min and generated 1,056,403 reads. The R10.3 flow cell was run until it obtained a comparative number of reads as the R9.4.1 library; this took 2 h 30 min of sequencing, and we obtained 1,060,000 reads (Supplementary Table S2).

We piped three datasets through Guppy for GPU basecalling on the laptop: one for the R9.4.1 dataset, and two from the R10.3 dataset, one “SR” for the same amount of reads generated as R9.4.1 (~1 million reads), and the other “ST” for the same length of sequencing time as R9.4.1 (~500,000 reads). We ran Fast and HAC basecalling for each of the three datasets on Guppy to obtain six basecalled datasets in total. We observed a 7–15 min difference between the Fast and HAC basecalling models, but did not observe any ostensible difference in basecalling times between R9.4.1 and R10.3_SR datasets (Supplementary Table S2). All nanopore fast5 read sets and corresponding basecalled fastq files have been deposited at the NCBI Sequence Read Archive under BioProject PRJNA657385 (SRR12466223–SRR12466228, and SRR12473542–SRR12473547).

While the Guppy results were fairly similar across the six datasets, we noted a more pronounced effect of the basecalling model on the number of MinION barcodes obtained. In general, datasets that were called using the Fast basecalling model resulted in a lower percentage of successfully demultiplexed reads (9–11% for Fast vs. 15–25% for HAC models). Low demultiplexing success rates were expected due to the intrinsically high raw read error rate [36], and our values were consistent with past studies [35,73]. The Fast datasets also consistently obtained a lower number of consolidated MinION barcodes than the HAC datasets (75–84 vs. 96–103; Table 1). The R10.3 dataset performed marginally better than R9.4.1 with respect to the final number of consolidated barcodes obtained (102–103 vs. 96). Remarkably, even with only half the read size of the R9.4.1_HAC dataset, the R10.3_HAC_ST dataset obtained even more barcodes than the former. We also noted only an increase in one more barcode in the R10.3_HAC_SR dataset, despite doubling the reads sequenced and increasing the run time three-fold.

### 3.4. MinION Barcode Assessment

**Figure 4:**
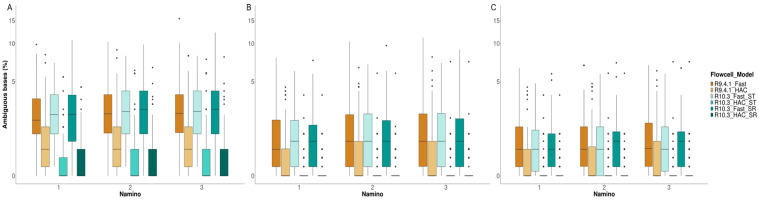
Percentage of ambiguous bases (%) for the three types of error-corrected MinION barcodes: (**A**) MAFFT + AA, (**B**) RACON + AA, and (**C**) consolidated. Colors represent the type of flow cell used (R9.4.1 or R10.3), along with the basecalling model applied (Fast or HAC). For the R10.3 datasets, we generated a subset for the same sequencing time (ST) as R9.4.1, and another dataset for the same number of reads (SR) as R9.4.1. Note that the y-axis was scaled using pseudo-log2 transformation for better representation.

Referenced against Illumina barcodes, MinION barcodes generated from the miniBarcoder pipeline scored high on accuracy (≥99%) regardless of the flow cell or basecalling model used (Table 2). While uncorrected barcodes (i.e., MAFFT and RACON barcodes) generated from the Fast basecalling model resulted in more gaps compared to the HAC model, the miniBarcoder pipeline was able to correct this disparity, such that all three types of error-corrected barcodes (MAFFT + AA, RACON + AA, and consolidated barcodes) have zero gaps across all flow cell and basecalling model datasets (Table 2). We did, however, note differences in barcode ambiguities remaining after error correction. In particular, we found that the basecalling model applied greatly influenced the proportion of remaining ambiguities in error-corrected MinION barcodes more so than the flow cell type. The HAC model was the superior model, and the resultant MinION barcodes consistently had fewer remaining ambiguities compared to the Fast basecalling barcodes (Figure 4). In fact, ~80% of the consolidated MinION barcodes from the R10.3_HAC datasets (ST and SR included) had 0% ambiguous bases.

We eventually selected the consolidated (namino2) barcodes from the R10.3_HAC_SR dataset as our primary MinION barcode set for two reasons. First, it was the dataset that yielded the highest number of MinION barcodes following contamination checks (*n* = 103). Second, it was also the dataset that did not have any remaining gaps and scored 100% sequencing accuracy when matched against Illumina references (Table 2). The namino3 dataset performed similarly well, but had a higher number of total ambiguous bases compared to the namino2 set (87 vs. 82). In addition, we further rescued two additional MinION barcodes (HS0019 and HS0157; see Section 2.6.) for the final dataset to yield a total of 105 MinION barcodes for this study (59% success).

### 3.5. DNA Barcodes and Species Diversity

Combining datasets of 116 Illumina and 105 MinION barcodes, including 74 overlapping barcodes, we obtained a total of 147 unique DNA barcodes from both sequencing platforms (81% success overall out of 182 samples). We derived 116 MOTUs at the 3% threshold, of which 93 were singletons. MOTU richness was stable across the 2–4% thresholds. When compared to the existing local Singapore barcode database [39], we found at least 70 novel MOTUs from this study. DNA barcodes generated in this study have been deposited at GenBank under accession numbers MT896212–MT896358 (Supplementary File S4).

## 4. Discussion

In this study, we assembled an in situ sequencing setup that comprised three main components: the suitcase-sized laboratory in the form of the BentoLab, the MinION handheld sequencer, and a laptop computer (Figure 1). Our proposed in situ sequencing workflow employed QE solution for thermal-based DNA extraction and tagged PCR on the BentoLab, before sequencing on the MinION and laptop. The laptop computer also served as an analysis terminal for basecalling and MinION barcode calling via miniBarcoder. We first tested all the protocols back at the laboratory, before conducting an in situ demonstration onboard a diving vessel moored at the Sisters’ Islands Marine Park, Singapore, on 15 July 2020 (Figure 2). We obtained 105 MinION barcodes, of which 19 were from samples obtained in the field. To our knowledge, the 31 samples and 19 DNA barcodes generated here represent one of the highest throughputs from published studies to date [20,21,31,32], with the entire sample-to-sequence workflow completed in under 9 h. In the following, we discuss our experiences with portable sequencing on the BentoLab and MinION in the form of three takeaways learnt from the entire process.

### 4.1. Takeaway #1: Portability and Productivity

While the MinION sequencer has undoubtedly been instrumental in making portable sequencing possible, the field-ready hardware has hitherto not co-evolved to keep pace with the sequencing technology. There thus remain certain logistical and operational limitations to carrying out DNA barcoding in situ as discussed recently [20,21,31,73]. One of the most consequential constraints is the sample throughput of portable laboratory equipment. Barcode amplification remains the most crucial yet time-limiting step in any DNA barcoding workflow, but only a handful of samples can be processed at any one given time due to the low capacity of existing portable laboratory equipment. This low scalability potentially limits its applicability and buy-in to portable sequencing. As such, we strongly advise new users to carefully consider the sequencing targets and objectives, so as to better plan around the field equipment and conditions to suit their own needs. In our case, we successfully expanded the processing capacity to 32 samples at any one time by using a one-step, heat-based DNA extraction method on the BentoLab. It is worth noting that the use of spin-column kits, as past studies have done [20,21,31], was impractical for our study given the 6-well configuration of the BentoLab centrifuge.

The overall higher throughput here would most certainly fortify existing barcoding capacities for species identification on expeditions or field courses [20,21,30,31,74], though unlikely to the extent where a “reverse workflow” of sorting specimens with DNA barcodes can be fully realized [35,47] given the relatively small number of samples that can be processed each time. Specifically, this increased barcoding capacity would be helpful for small-scale operations involving randomly selected samples, or where on-site testing is preferred but laboratory capabilities are not available, particularly in biosecurity, wildlife conservation genetics, and even food safety [70,75,76,77,78]. While the BentoLab is slightly bulkier compared to the miniPCR, our entire sequencing system would still fit in a backpack and not require much space to set up and deploy.

### 4.2. Takeaway #2: Operational Costs

One of the strengths of second- and third-generation sequencers is the ability to reduce sequencing costs via sample multiplexing onto a flow cell [15]; the greater the number of samples, the lower the resultant cost of each barcode. MinION barcodes can cost as low as ~USD 0.35 each when multiplexing ~3000 samples per MinION flow cell [35], though such a volume is unlikely to be achieved in a field setting [32,53]. Users thus need to bear in mind that there are financial trade-offs with the lower throughput for portable sequencing. For our entire sample set (188 amplicons), we estimated each MinION barcode to cost ~USD 6.50, whereas if we barcoded only the field collections (32 amplicons), MinION barcoding would have cost USD 33.70 per barcode (Supplementary Table S3). The latter is nearly double the cost of USD 18 per regular Sanger barcode [15]. Our workflow has sought to keep molecular costs low by using QE-based DNA extraction, which we estimated to be USD 0.60 a sample. It is slightly more costly than the Chelex resin (USD 0.17 per sample), but still considerably cheaper than other proprietary extraction kits sold by Qiagen or Biomeme (USD 3 and USD 15, respectively, per sample) [73]. The thermal-based DNA extraction method complemented the 32-well capacity of the BentoLab and was instrumental in increasing our throughput.

For gene amplification, it was cheaper to use tagged primers [35] which allowed for more samples to be multiplexed onto the flow cell, compared to ONT’s native barcoding expansion kit for a maximum of 96 samples. The former also saved us an additional barcode ligation step during library preparation. One other fruitful way to reduce sequencing costs is to use the lower-throughput Flongle flow cells [31,70], which are estimated to cost USD 10 per DNA barcode on a Flongle multiplexed with 96 samples [31]. We did not test the Flongle for this study as the R10.3 chemistry is presently limited to MinION flow cells. Nevertheless, the field of on-site nanopore barcoding is rapidly growing, and researchers are increasingly finding creative ways to reduce costs, such as 3D-printing of centrifuges to complement spin-column kit extractions [79,80,81]. We expect that as novel techniques emerge and technologies are refined, the cost of in situ nanopore barcoding is likely to fall even more in the near future.

### 4.3. Takeaway #3: Flow Cell Chemistry and Basecalling Model

This study also investigated how flow cell chemistry and basecalling models affected sequencing accuracy and barcode quality by analyzing six different datasets. We observed that the default HAC (high-accuracy) basecalling model was superior. HAC datasets attained higher demultiplexing success compared to Fast datasets (Table 1) due to the improved basecalling accuracy in the HAC model. In all instances, datasets that employed the HAC model resulted in more barcodes overall than the Fast datasets (Table 1). We also noted ostensible differences in barcode quality, where HAC-produced barcodes had fewer persisting ambiguities across all three types of error-corrected barcodes (Figure 4). Moreover, we did not observe significant time savings from applying the Fast basecalling model (Supplementary Table S2). There appears to be no compelling reason to adopt the Fast model and we recommend that users adhere to the default HAC model for basecalling.

There was a final difference of just one barcode between the ST (R10.3 with the same sequencing time as R9.4.1) and SR (R10.3 with the same number of reads as R9.4.1) datasets. Prolonging the sequencing time improved coverage but not the final barcode tally (Table 1). Indeed, a run time of ~50 min on R10.3 was sufficient to capture the full range of sample diversity with 188 amplicons, and there was no evidence to suggest that the raw read count impacted the final barcode tally in any way. In fact, the R10.3_HAC_ST dataset resulted in more consolidated barcodes than the R9.4.1_HAC dataset (102 vs. 96) with only half the number of raw reads generated. Fewer raw reads processed translated to faster Guppy basecalling times (~2× faster; Supplementary Table S2) and would be especially important for field sequencing workflows like ours where rapid turnover is key.

The pairing of the HAC model with the R10.3 flow cell chemistry resulted in the highest quality of MinION barcodes for this study. This finding is evident in how all corrected barcodes had no internal gaps, scored near perfect sequencing accuracy (≥99.87%; Table 2), and a large majority (~80%) had zero ambiguities post-correction (Figure 4). In contrast, only ~52% and ~63% of R9.4.1_HAC barcodes from this study and an earlier study [37], respectively, were free of ambiguities. N-coded bases are typically inserted during amino acid correction to resolve frameshifts caused by sequencing errors in homopolymeric regions [36]. The observed increase in samples having zero ambiguous bases points to an improved homopolymer resolution with the R10.3 chemistry. This marked improvement in R10.3 sequencing chemistry is a welcome development and paves the way for furthering nanopore sequencing applications such as DNA metabarcoding [82,83,84,85]. Error-prone reads from the R9 chemistry make it challenging to assign taxonomy [53], and previous studies have resorted to complex laboratory procedures [84] or reference-based polishing [82] to negate these sequencing errors. One study tested the R10.3 chemistry for nanopore metabarcoding, but the only comparisons made to R9.4.1 chemistry were in terms of read coverage and read size distribution; no assessments were made on sequencing accuracy [83]. Given the improved DNA barcode performance noted in this study, we believe similar positive knock-on effects for nanopore metabarcoding are to be expected.

## 5. Conclusions

Major advancements in sequencing technology, such as the release of ONT’s handheld MinION sequencer, have made portable sequencing possible, and numerous studies have since emerged to advance this field. However, field-based barcoding capacity remains limited to small sample sizes. Here, we expand upon existing capabilities by combining the use of BentoLab with the MinION. The BentoLab boasts a 32-well thermocycling capacity, and is suited for a thermal-based DNA extraction method like QE. Our proof-of-principle demonstration out at sea generated 105 MinION barcodes, 19 of which were from samples processed immediately after collection. To date, our field collection of 31 specimens represents one of the largest sets of samples processed in situ. We also took the opportunity to test the newly released R10.3 flow cell for DNA barcoding, and report that the error-corrected barcodes scored high on sequencing accuracy, had no gaps, and showed an improved homopolymer resolution compared to the existing R9.4.1 chemistry. Collectively, the Illumina and MinION sequencing runs here have contributed 147 more barcodes toward efforts to grow the local biodiversity knowledge database. Our in situ sequencing workflow is thus viable and joins a growing myriad of related developments aimed at advancing portable DNA barcoding capabilities, raising throughput and lowering costs as the field progresses.

## Figures and Tables

**Table 1:** MinION reads and barcodes obtained among datasets for each flow cell and basecalling model. The number of error-corrected barcodes was the same regardless of the namino setting used. Clean consolidated barcodes refer to remaining number of consolidated barcodes post-contamination check.

	R9.4.1_Fast	R9.4.1_HAC	R10.3_Fast_ST	R10.3_HAC_ST	R10.3_Fast_SR	R10.3_HAC_SR
**Basecalled reads**	1,056,403	1,056,403	512,000	512,000	1,060,000	1,060,000
**Demultiplexed (%)**	115,833 (11.0)	161,376 (15.3)	50,203 (9.8)	109,955 (21.5)	121,579 (11.5)	264,501 (25.0)
**Read depth per sample**	11–36,925	11–49,990	10–2517	11–5086	10–6037	10–12,221
**MAFFT / <1% Ns-filter**	125/101	126/111	115/92	121/114	122/101	128/117
**RACON**	101	111	92	114	101	117
**MAFFT+AA**	97	110	90	113	99	115
**RACON+AA**	98	110	91	113	100	115
**Consolidated**	86	104	83	111	92	113
**Consolidated (Clean)**	79	96	75	102	84	103

**Table 2:** Sequencing accuracy (A) and gaps (G) observed when comparing the overlapping number (*N*) of MinION barcodes with Illumina references.

	R9.4.1_Fast			R9.4.1_HAC			R10.3_Fast_ST			R10.3_HAC_ST			R10.3_Fast_SR			R10.3_HAC_SR		
**Barcode**	*N*	G	A (%)	*N*	G	A (%)	*N*	G	A (%)	*N*	G	A (%)	*N*	G	A (%)	*N*	G	A (%)
**MAFFT Ns-filter**	65	198	99.9800	74	99	100.0000	62	202	99.9843	76	31	99.9958	69	233	99.9812	77	33	100.0000
**RACON**	65	119	99.9801	74	50	100.0000	62	121	99.9480	76	7	100.0000	69	125	99.9673	77	9	99.9834
**MAFFT+AA (namino1)**	62	0	99.9428	73	1	99.9516	61	0	99.9315	75	0	99.9914	68	4	99.9149	76	0	99.9916
**MAFFT+AA (namino2)**	62	0	99.9479	73	1	99.9736	61	0	99.9525	75	0	99.9957	68	2	99.9479	76	0	100.0000
**MAFFT+AA (namino3)**	62	2	99.9791	73	1	99.9780	61	0	99.9524	75	0	99.9957	68	4	99.9668	76	0	100.0000
**RACON+AA (namino1)**	63	1	99.9387	73	0	99.9780	61	4	99.8735	75	0	99.9914	68	0	99.9339	76	0	99.9620
**RACON+AA (namino2)**	63	1	99.9488	73	0	99.9912	61	4	99.9101	75	0	100.0000	68	0	99.9479	76	0	99.9831
**RACON+AA (namino3)**	63	1	99.9589	73	0	99.9912	61	5	99.9204	75	0	100.0000	68	0	99.9525	76	0	99.9831
**Consolidated (namino1)**	55	0	99.9532	70	0	99.9679	58	0	99.9003	73	0	99.9912	64	0	99.9599	74	0	99.9913
**Consolidated (namino2)**	55	0	99.9648	70	0	99.9862	58	0	99.9223	73	0	99.9956	64	0	99.9649	74	0	100.0000
**Consolidated (namino3)**	55	0	99.9707	70	0	99.9862	58	0	99.9333	73	0	99.9956	64	0	99.9648	74	0	100.0000

## Supplementary Materials

Supplementary materials can be found at https://www.mdpi.com/2073-4425/11/10/1121/s1. File S1: Sample demultiplexing information for miniBarcoder; File S2: UNIX script used to automate the miniBarcoder barcode calling; File S3: Illumina-generated reference barcodes for validation; File S4: List of specimens barcoded in this study, including voucher numbers and GenBank accession numbers; Table S1: Cost evaluation of reverse barcoding primers; Table S2: GPU specifications, Guppy settings, and run statistics; Table S3: Estimated costs for portable DNA barcoding with the BentoLab and MinION.
