# Supplementary material for: Takeaways from Mobile DNA Barcoding with BentoLab and MinION
Source: Genes (Basel). 2020 Sep 24;11(10):1121. doi: 10.3390/genes11101121 (PMC7598690; doi:10.3390/genes11101121)
Supplement: Supplementary file 1 [file genes-11-01121-s001.zip › 20200923_Supplementary/SuppTables.docx]

Supplementary Table S1. Cost evaluation of the reverse barcoding primers jgHCO2198 and LoboR1. Sequence differences are highlighted in red. Prices listed are sourced from Integrated DNA Technologies (IDT), Singapore.

| **Primer** | **Reference** | **Primer Sequence (5’ 🡪 3’)** | **Primer cost (USD)** |
| --- | --- | --- | --- |
| jgHCO2198 | Geller et al., 2013 | TAI ACY TCI GGR TGI CCR AAR AAY CA | $34.15 |
| LoboR1 | Lobo et al., 2013 | TAA ACY TCW GGR TGW CCR AAR AAY CA | $8.62 |

References:

Geller, J.; Meyer, C.; Parker, M.; Hawk, H. Redesign of PCR primers for mitochondrial cytochrome c oxidase subunit I for marine invertebrates and application in all-taxa biotic surveys. *Molecular Ecology Resources*. **2013**, *13*, 851–861.

Lobo, J.; Costa, P.M.; Teixeira, M.A.L.; Ferreira, M.S.G.; Costa, M.H.; Costa, F.O. Enhanced primers for amplification of DNA barcodes from a broad range of marine metazoans. *BMC Ecology*. **2013**, *13*, 34.

Supplementary Table S2. GPU specifications for Guppy basecalling, and the time taken for each dataset. Datasets are labelled “<flowcell>_<basecalling model>”. R10.3 datasets appended with “SR” have approximately same number of reads generated with R9.4.1, while datasets appended with “ST” were sequenced for the same amount of time as R9.4.1.

| NVIDIA GeForce RTX2060   - 7.5 Compute Capability - 6 GB RAM   Driver v.418.56  CUDA Toolkit v10.1  Guppy 4.0.14+8d3226e | | | | Settings enabled during GPU basecalling:  *--min_qscore 0*  *--gpu_runners_per_device 2*  *--chunks_per_runner 512*  *--chunk_size 2000* | | | |
| --- | --- | --- | --- | --- | --- | --- | --- |
|  | **R9.4.1_Fast** | **R9.4.1_HAC** | **R10.3_Fast_ST** | | **R10.3_HAC_ST** | **R10.3_Fast_SR** | **R10.3_HAC_SR** |
| No. fast5 files processed | 265 | 265 | 128 | | 128 | 265 | 265 |
| No. reads generated | 1,056,403 | 1,056,403 | 512,000 | | 512,000 | 1,060,000 | 1,060,000 |
| Rate (sample/s, 1e^6^) | 1.15 | 0.91 | 2.72 | | 2.12 | 2.98 | 2.40 |
| Caller time (min) | 53.9 | 68.1 | 26.3 | | 33.2 | 55.3 | 67.7 |

Supplementary Table S3. Estimated costs for in situ DNA barcoding, from DNA extraction to MinION barcoding. Costs were estimated based on total amplicon size for the entire study (*n* = 188; FULL), and for the field sampling alone (*n* = 32; FIELD). Primers, clean-up reagents and consumables were omitted as they did not greatly affect the price of the barcode.

| **Package Cost (USD)** | **Per Sample Cost, FULL (USD)** | **Per Sample Cost, FIELD (USD)** |
| --- | --- | --- |
| $138.90 per QuickExtract^TM^ Lucigen, 5ml (for 250 reactions) | $0.60 | $0.60 |
| $296.72 per GoTaq® Green Master Mix (1,000 reactions) | $0.30 | $0.30 |
| $900 per flow cell (used 1)  $599 per Ligation Sequencing Kit (6 preps, used 1)  $1,204.33 per NEBNext ONT Companion Kit (24 preps, used 1) | $5.59 (188 amplicons) | $32.80 (32 amplicons) |
| **TOTAL** | $6.49 | $33.70 |
